# Lectin Pathway Mediates Complement Activation by SARS-CoV-2 Proteins

**DOI:** 10.3389/fimmu.2021.714511

**Published:** 2021-07-05

**Authors:** Youssif M. Ali, Matteo Ferrari, Nicholas J. Lynch, Sadam Yaseen, Thomas Dudler, Sasha Gragerov, Gregory Demopulos, Jonathan L. Heeney, Wilhelm J. Schwaeble

**Affiliations:** ^1^ Department of Veterinary Medicine, School of Biological Sciences, University of Cambridge, Cambridge, United Kingdom; ^2^ Department of Microbiology and Immunology, Faculty of Pharmacy, Mansoura University, Mansoura, Egypt; ^3^ Omeros Corporation, Seattle, WA, United States

**Keywords:** complement system, lectin pathway, SARS-CoV-2, COVID-19, innate immunity

## Abstract

Early and persistent activation of complement is considered to play a key role in the pathogenesis of COVID-19. Complement activation products orchestrate a proinflammatory environment that might be critical for the induction and maintenance of a severe inflammatory response to SARS-CoV-2 by recruiting cells of the cellular immune system to the sites of infection and shifting their state of activation towards an inflammatory phenotype. It precedes pathophysiological milestone events like the cytokine storm, progressive endothelial injury triggering microangiopathy, and further complement activation, and causes an acute respiratory distress syndrome (ARDS). To date, the application of antiviral drugs and corticosteroids have shown efficacy in the early stages of SARS-CoV-2 infection, but failed to ameliorate disease severity in patients who progressed to severe COVID-19 pathology. This report demonstrates that lectin pathway (LP) recognition molecules of the complement system, such as MBL, FCN-2 and CL-11, bind to SARS-CoV-2 S- and N-proteins, with subsequent activation of LP-mediated C3b and C4b deposition. In addition, our results confirm and underline that the N-protein of SARS-CoV-2 binds directly to the LP- effector enzyme MASP-2 and activates complement. Inhibition of the LP using an inhibitory monoclonal antibody against MASP-2 effectively blocks LP-mediated complement activation. FACS analyses using transfected HEK-293 cells expressing SARS-CoV-2 S protein confirm a robust LP-dependent C3b deposition on the cell surface which is inhibited by the MASP-2 inhibitory antibody. In light of our present results, and the encouraging performance of our clinical candidate MASP-2 inhibitor Narsoplimab in recently published clinical trials, we suggest that the targeting of MASP-2 provides an unsurpassed window of therapeutic efficacy for the treatment of severe COVID-19.

## Introduction

Coronaviruses (CoVs) are single-stranded RNA viruses causing life threatening respiratory infection in humans and other species. The CoV genome encodes four main structural proteins, spike (S), membrane (M), envelope (E), and nucleocapsid (N), as well as other accessory proteins that facilitate replication and entry into cells. The transmembrane bound spike protein (S), consists of two subunits S1 and S2 that cover the surface of CoVs and serve as receptor binding entry proteins for infection. The nucleocapsid protein complexes with the viral RNA and plays a major role in viral replication as well as viral pathogenesis. M and E are two transmembrane proteins, which are responsible for viral assembly (1). In 2019, a pandemic respiratory infection caused by corona virus was reported and identified as coronavirus disease 2019 (COVID-19), the etiological agent of which is a β-coronavirus called severe acute respiratory syndrome coronavirus 2 (SARS-CoV-2). The clinical manifestation of SARS-CoV-2 infection include fever, cough, fatigue, myalgia, and pneumonia, that may develop into acute respiratory distress syndrome (ARDS), necessitating respiratory support, as well as disseminated intravascular coagulopathy and kidney failure (2, 3).

The complement system (CS) is an integral part of the innate and the adaptive immune systems. The CS is composed of more than 30 plasma and cell-resident components that form a first defence-line against infection and provides an essential scavenger system to eliminate injured, apoptotic or aberrant cells. Complement activation products modulate inflammation and direct the innate and the adaptive immune response. The CS is activated *via* three pathways, which funnel into a shared terminal activation route. The classical pathway (CP) is initiated through the binding of a recognition subcomponent, specifically complement component 1q (C1q). Two C1q-associated serine protease zymogens, C1r and C1s, form a C1s-C1r-C1r-C1s hetero-tetramer, which sits within the calix of the C1q macromolecule. The C1r/C1s zymogen complex is converted into active form when at least two arms of the C1q macromolecule bind to the Fc region of immune complexes. Activation of C1s leads to the cleavage of C4 to C4a and C4b, with the latter binding to C2. C4b-bound C2 is then cleaved by C1s to create the C3 convertase C4b2a, which cleaves the abundant complement component C3 into the anaphylatoxin C3a and the major fragment C3b (4). The Lectin pathway (LP) is initiated by multimolecular pattern-recognition complexes that bind to immune complexes and pathogen-associated molecular patterns (PAMPs). Six different LP recognition subcomponents can form LP activation complexes by binding dimers of three different mannan-binding lectin-associated serine proteases (i.e., MASP-1, MASP-2 and MASP-3). The recognition subcomponents comprise multimers of homotrimeric chains, which can bind directly to their cognate ligands present on pathogens, or to aberrant glycosylation patterns on apoptotic, necrotic, malignant, or damaged host cells (5, 6). The LP recognition subcomponents in humans are: mannan-binding lectin-2 (MBL-2), collectin-11 (CL-11), heterocomplexes of CL-11 and CL-10 and three different ficolins (ficolins 1, 2, and 3), two of which can also form heterocomplexes (ficolins 2 and 3). MASP-2 is the key enzyme of the LP; only MASP-2 can cleave C4 efficiently, whereas both MASP-2 and MASP-1 can cleave C2. In the absence of MASP-2, complement can no longer be activated by the LP activation route because the C3 and C5 convertase complexes C4b2a and C4b2a (C3b)n cannot be formed (7, 8). In addition, MASP-2 was shown to cleave C3 directly, forming a novel C4-bypass activation route. This C4-bypass route was shown to be important in the innate immune defence (9). The alternative pathway (AP) fulfils its surveillance function through a constant low-rate activation (C3 tick-over) and provides an efficient amplification loop of C3 activation. The C3 activation product C3b can bind to zymogen complement factor B (FB), forming a complex that can in turn convert more C3 into C3a and C3b if C3b-bound FB is cleaved by a serine protease called FD (10).

Complement activation was reported to be associated with development of acute respiratory distress syndrome (ARDS) and respiratory failure during viral pneumonia (11, 12). A direct link between complement activation and pathogenesis of Corona virus infection was established using C3-deficient mice infected with SARS-CoV. C3-deficient mice showed significantly less severe respiratory inflammation, decreased infiltration of neutrophils and inflammatory monocytes, and lower levels of cytokines and chemokines in both the lungs and sera compared to wild-type control mice (13). The involvement of complement-mediated pathology and lung injury during SARS-CoV-2 infection was revealed by a histopathological study of post-mortem biopsies taken from COVID-19 patients. The presence of thrombotic microangiopathies (TMAs) and the deposition of complement activation products, including C5b-9, C3d, C4d and the LP effector enzyme MASP-2 implied the involvement of LP and CP activation in severe COVID-19 (14). In this study, we address the involvement of the lectin activation pathway of complement in the response against recombinant SARS-CoV-2 proteins and which can trigger activation of the complement system.

## Materials and Reagents

Recombinant S and N proteins of SARS-CoV-2 expressed in mammalian cell lines were purchased from R & D systems, UK. The pEVAC plasmids expressing the coding sequences for S protein were kindly provided by DIOSynVax Ltd Cambridge, UK. Recombinant truncated MASP-2, containing the 2 CCP domains and the serine protease domain, was expressed previously described (9).

HG4, a monospecific fully humanised antibody against MASP-2 that inhibits LP-mediated C4 cleavage was kindly provided by Omeros Corporation, Seattle, USA. Pre-pandemic, non-immune NHS, was pooled from 4 healthy donors. The mean levels of key lectin pathway components in the pool were: MBL, 1.43µg/ml; FCN2, 2.9µg/ml; CL-11, 0.39µg/ml; and MASP-2, 0.4µg/ml.

### Transfection of HEK 293T Cells

HEK 293T cells were cultured in Dulbecco’s Modified Eagle’s Medium (DMEM) supplemented with 10% Foetal bovine serum albumin (FBS), 2 mM glutamine and 10 U/mL penicillin, 10 μg/mL streptomycin (Gibco). Cells were maintained in a CO_2_ incubator at 37°C. HEK 293T cells were seeded in 6-well plates with cell density of 1×10^6^ cells/mL. Next day, cells were transfected with 1 μg of plasmid DNA for each well using the Fugene transfection kit (Promega) according to the manufacturer’s protocol. Cells transfected with empty pEVAC vector were used as a control. 48 hours after transfection, cells were harvested for flow cytometer analysis.

### FACS Analysis

Transfected HEK 293T cells were washed twice using Hank’s balanced salt solution with C^2+^ and Mg^2+^ (HBSS^++^) and resuspended in HBSS^++^ to a final concentration of 10^7^ cell/mL. 10^6^ cells were opsonised with 2.5% NHS in HBSS^++^ for 30 minutes at 37°C with or without 100 nM of HG4. Cells transfected with empty vector were used as a negative control. After opsonization, cells were washed twice with HBSS^++^ buffer, and bound C3b was detected using FITC-conjugated rabbit anti-human C3c (Dako). Fluorescence intensity was measured with The Attune NxT Flow Cytometer (Invitrogen).

### Solid Phase Binding Assays

Nunc MaxiSorp microtiter ELISA plates were coated with 10 μg/mL of purified recombinant SARS-CoV-2 proteins S and N in coating buffer (10 mM Tris-HCl, 140 mM NaCl, pH 7.4). Control wells were coated with 10 μg/mL mannan (a control for MBL binding), 10 μg/mL zymosan (a control for CL-11 binding) or 10 μg/mL N-acetylated BSA (a control for L-ficolin binding). Immune complexes formed by incubation of BSA with rabbit anti-BSA were prepared. The ELISA plates were coated with 1 μg/mL BSA-anti-BSA immune complex as a control ligand for C1q binding. The following day, wells were blocked for 2 hours at room temperature with 250 μL of 1% (w/v) BSA in TBS buffer (10 mM Tris-HCl, 140 mM NaCl, pH 7.4), then washed three times with 250 μL of TBS with 0.05% Tween 20 and 5 mM CaCl_2_ (wash buffer). Serial dilutions of serum in 100 μL of wash buffer were added to the wells and the plates were then incubated for 90 minutes at room temperature. Plates were washed as above and bound proteins were detected using rabbit anti-human L-ficolin, mouse anti-human CL-11 or mouse anti-human MBL mAbs. HRP conjugated goat anti-rabbit IgG followed by the colorimetric substrate (15).

### Complement Deposition Assays

To measure C3 and C4 activation, Nunc MaxiSorp microtiter plates were coated with 100 μL of 10 μg/mL mannan (Promega), or 100 μL of 10 μg/mL SARS-CoV-2 proteins in coating buffer. After overnight incubation, wells were blocked with 1% BSA in TBS then washed with wash buffer. Serum samples were diluted in BBS (4 mM barbital, 145 mM NaCl, 2 mM CaCl_2_, 1 mM MgCl_2_, pH 7.4), starting at 5%, then added to the plates and incubated for 1.5 hours at 37°C. The plates were washed again, and bound C3b or C4b were detected using rabbit anti-human C3c (Dako) or rabbit anti-human C4c (Dako) followed by HRP conjugated goat anti-rabbit IgG followed by the colorimetric substrate TMB (15).

### Complement Inhibition Assay

The activity of HG4 against MASP-2 was tested using C4b deposition assay. 2.5% NHS containing different concentrations of Hg4 in BBS were added to an ELISA plate coated with mannan as previously described. Control wells received no antibodies. The plate was incubated at 37°C for 15 min, then washed. Bound C4b were detected using rabbit anti human C4c (Dako, Denmark) followed by an HRP conjugated goat anti- rabbit IgG (Sigma, USA). Bound antibody was detected using the Colorimetric substrate TMB.

### MASP-2 Binding Assay With SARS-CoV-2 Proteins

An ELISA plate was coated with 100 μL of 10 μg/mL SARS-CoV-2 proteins in coating buffer. Wells were blocked with 1% BSA in TBS then washed with wash buffer. Wells coated with BSA only were used as a negative control. Serial concentrations of recombinant MASP-2 in BBS, starting from 1μg/mL, were added to the plate and incubated at room temperature. After 1 hour, the plate was washed and MASP-2 binding to SARS-CoV-2 proteins was detected using monoclonal antibodies against MASP-2 followed by HRP-conjugated rabbit anti-human IgG and the chromogenic substrate ELISA Colorimetric TMB Reagent (Sigma). In a parallel experiment, 1μg of rMASP-2 in 100 μL BBS was incubated with wells coated with SARS-CoV-2 proteins for 1 hour at 37°C. After three washing steps using wash buffer, 2.5 μg of purified C4 (Comptech, USA) in 100 μL BBS were added to each well. Purified C4 added to wells coated with BSA was used as a negative control. After 1-hour incubation at 37°C, supernatants were collected from each well and boiled with 4X SDS loading dye. C4 cleavage mediated *via* MASP-2 was detected using SDS-PAGE and Coomassie bule staining under reducing conditions.

## Results

### Recognition Molecules of LP Bind to SARS-CoV-2 Proteins

**Figure 1:**
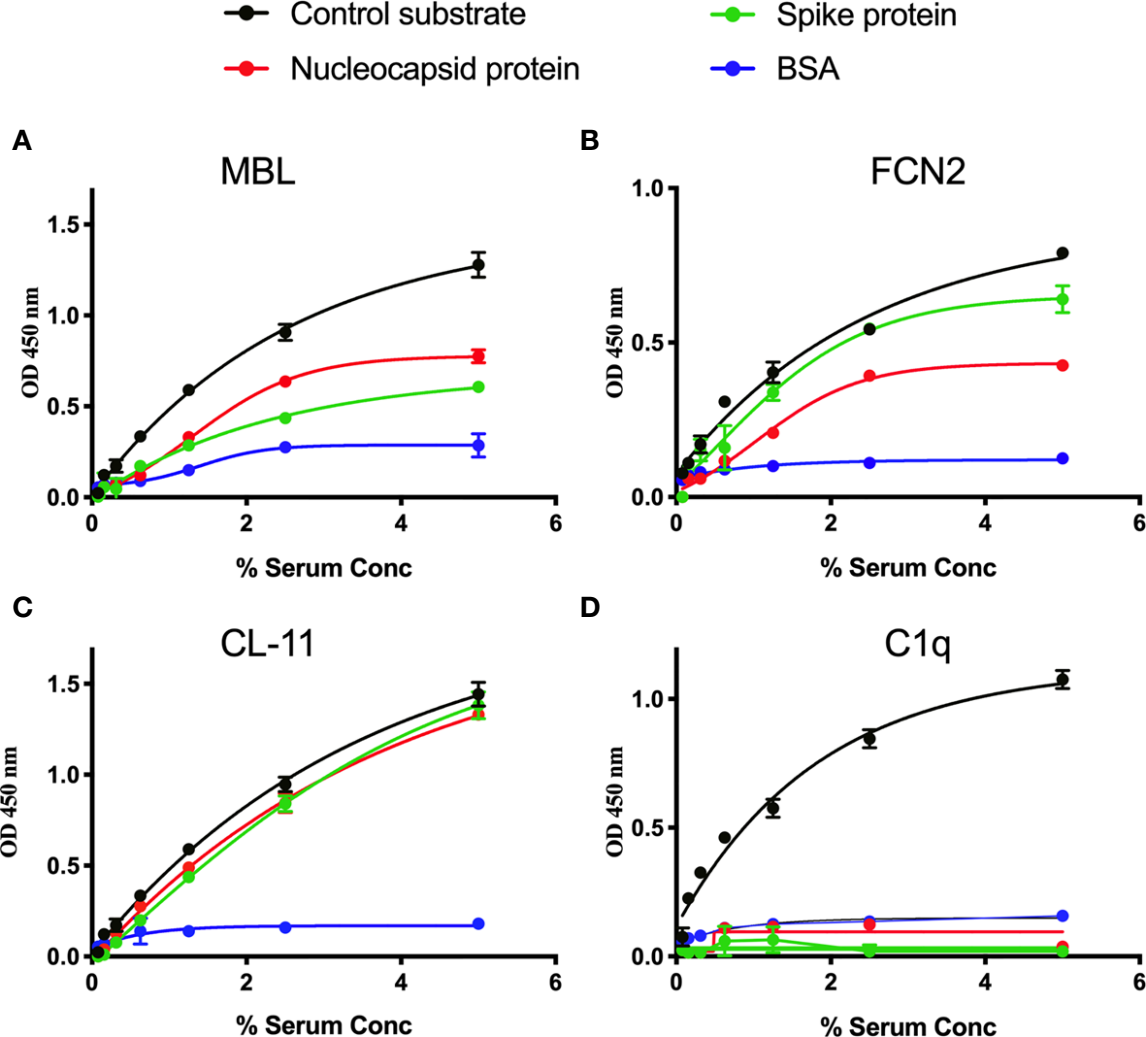
Binding of LP recognition molecules and C1q to SARS-CoV-2 proteins. A microtitre ELISA plate was coated with either S, N or control ligands (mannan for MBL, N-acetyl BSA for FCN2, zymosan for CL-11 or immune complexes for C1q). Following incubation with blocking buffer and washing steps, serial dilutions of NHS, starting at 5% were added to detect binding of LP recognition molecules and C1q from NHS to SARS-CoV-2 proteins. Human MBL **(A)**, FCN2 **(B)**, Cl-11 **(C)** and C1q **(D)** were assayed by ELISA. A significant binding of LP recognition molecules to S and N proteins was clearly observed. No C1q binding to any of the viral proteins was detected. Results are means of duplicates ± SD.

A series of solid-phase binding ELISA were performed to identify LP recognition molecules present in NHS that bind SARS-CoV-2 proteins. MBL, FCN2 and CL-11 bind to S and N proteins, indicating possible activation of the complement system *via* the LP. Interestingly, no C1q binding with SARS-CoV-2 proteins was observed using non-immune NHS, suggesting that the classical pathway is not activated in the absence of specific antibody (**Figure 1**).

### The Lectin Pathway Drives Complement Deposition on SARS-CoV-2

**Figure 2:**
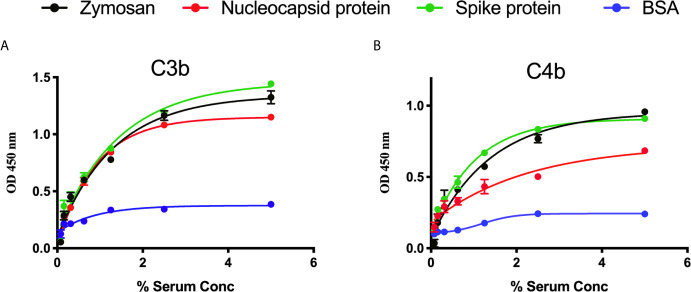
Detection of C3b and C4b deposition on SARS-CoV-2 proteins. An ELISA plate was coated with SARS-CoV-2 proteins (S or N) or zymosan and incubated with serial dilutions of NHS (starting at 5%) for 1 h at 37°C. C3b or C4b deposition were detected using antibodies against human C3c or C4c. High levels of C3b **(A)** and C4b **(B)** deposition were observed on surface immobilised SARS-CoV-2 proteins.

We measured complement C3b and C4b deposition on SARS-CoV-2 proteins immobilised on microtiter plates. When serial dilutions of pre-pandemic NHS were incubated on the plates, there was a dose-dependent and saturable deposition of C3b and C4b, indicative of LP activation, and comparable with the control substrates. Essentially no C3b or C4b deposition was detected on wells that were just blocked with BSA (p<0.01, 2-way ANOVA vs. the control) (**Figures 2A, B**).

### HG4 Inhibits LP Mediated C4b Deposition

**Figure 3:**
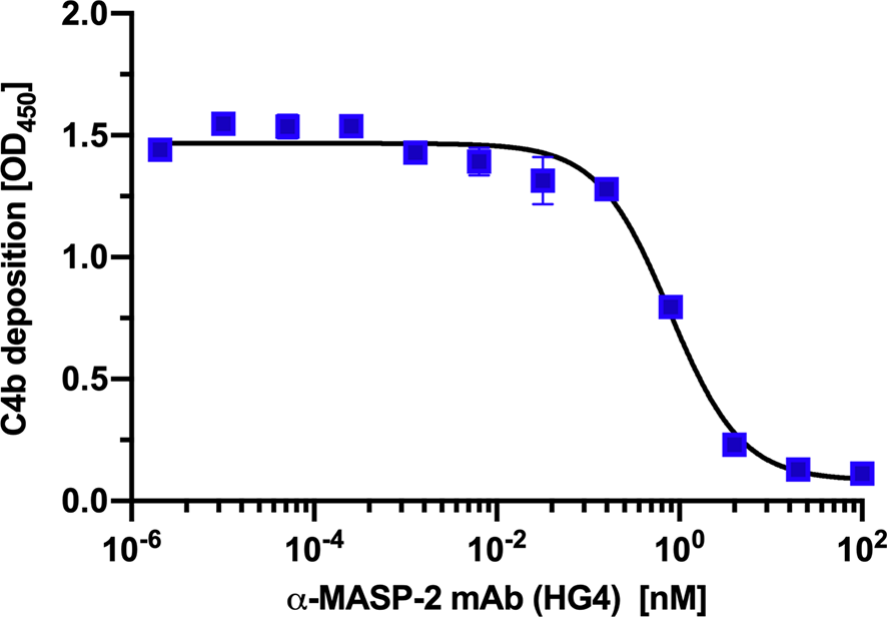
HG4 inhibits lectin pathway mediated C4b deposition. A microtiter ELISA plate was coated with mannan and blocked with BSA. Different concentrations of HG4 antibody were mixed with 2% NHS and incubated on the plate. Bound C4b was detected using anti C4c antibodies.

The ability of the fully humanised monoclonal antibody HG4 to inhibit LP functional activity was assessed using a C4b deposition inhibition assay. Our results showing that HG4 significantly inhibits LP functional activity with an IC50 around 0.74 nM (**Figure 3**).

### MASP-2 Binds Directly to N Protein and Promotes MASP-2-Mediated C4 Cleavage

**Figure 4:**
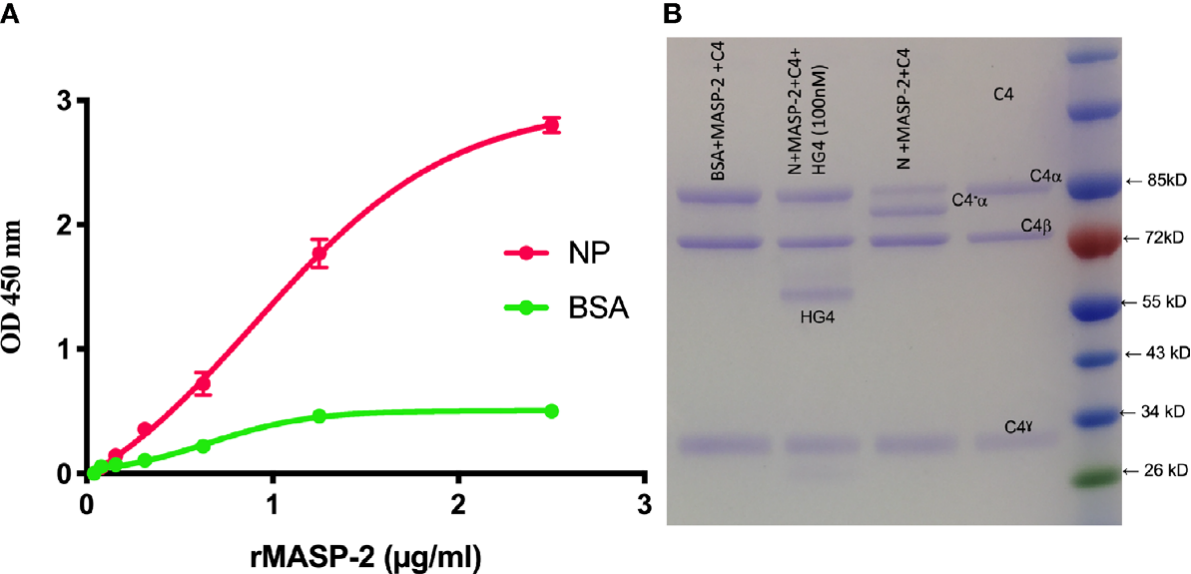
MASP-2 binds directly to SARS-Cov-2 N-protein and mediates complement C4 activation. **(A)** Microtiter plates were coated with 2.5μg/well N-protein or BSA as a control. Residual binding sites were blocked using 1% BSA. Serial dilutions of rMASP-2 were added, and binding was detected using an anti-MASP-2 mAb. MASP-2 bound to the NP protein in a concentration-dependent and saturable manner **(A)**. **(B)** In a parallel experiment, 1μg of rMASP-2 in barbital buffered saline (BBS) was added to wells coated with NP or BSA. After 1 hr at 37°C, wells were washed and 1μg of purified human C4 was added to each well. After 1 hr incubation at 37°C, the supernatant was collected and separated on SDS-PAGE. The results showed that rMASP-2 binds directly to NP and cleaves C4. Addition of HG4 (a mAb that inhibits MASP-2) inhibited MASP-2 mediated C4 cleavage. Purified C4 was run on the gel as a control.

The ability of MASP-2 to bind directly to SARS-CoV-2 N protein was tested using ELISA. A significant binding of rMASP-2 to N protein was detected. To further confirm the functional significance of this finding, ELISA wells coated with N-protein or BSA were incubated with 1μg of rMASP-2 for 1 hour at 37°C. After washing, 2.5 μg of purified C4 in BBS was then added to the wells and incubated at 37°C. After 1 hour, the supernatant was removed from the wells and the degree of C4 cleavage was analysed using SDS-PAGE. C4 cleavage was observed when purified C4 was incubated with N protein and rMASP-2 but not with BSA and rMASP-2. This experiment clearly showed that MASP-2 binds to N protein and promotes LP-mediated C4 cleavage. Interestingly, inhibition of MASP-2 activity using HG4 completely blocks LP-mediated C4 cleavage (**Figure 4**).

### Evaluation of HEK 293 T Cells Expressing SARS-CoV-2 Surface Proteins as a Model to Detect COVID-19-Related Complement Activation

**Figure 5:**
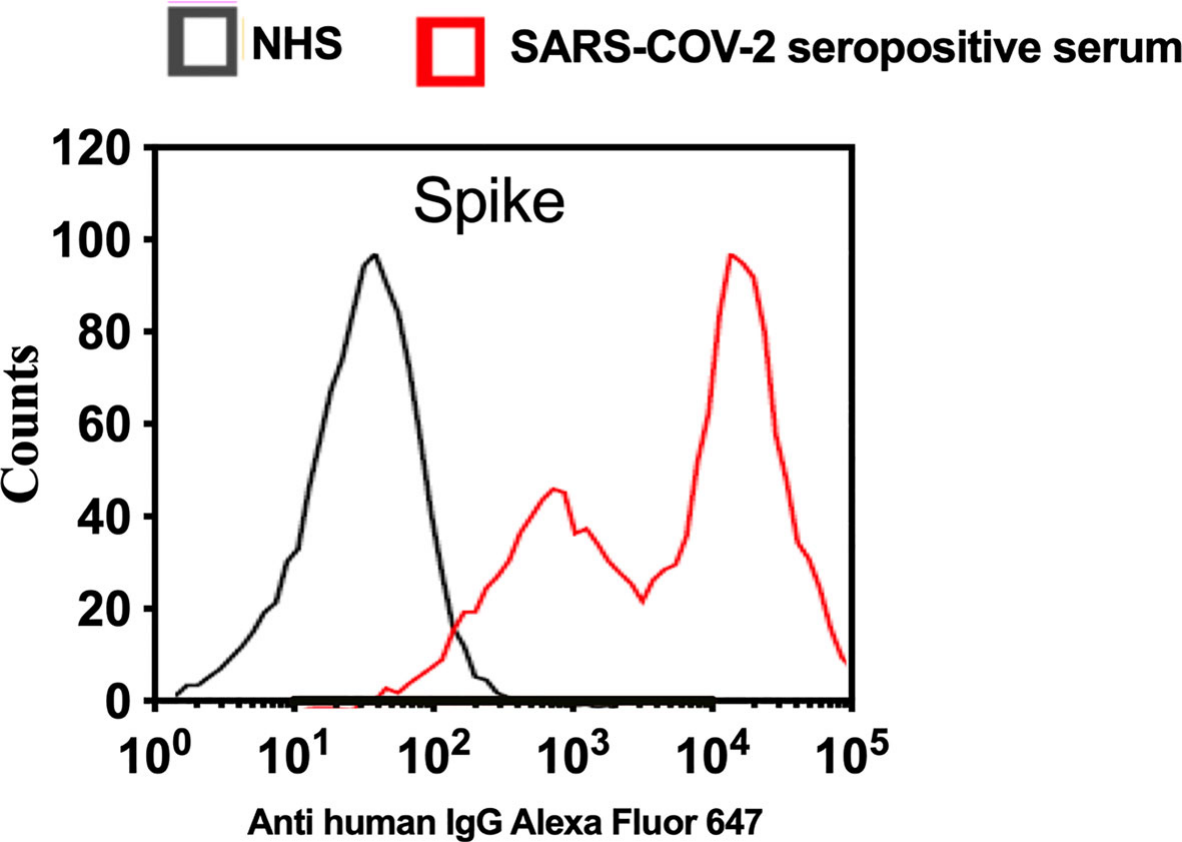
Transfection of HEK 293T promotes high levels of expression of SARS-CoV-2 surface proteins. The expression levels of S protein on the surface of HEK 293 T cells were measured using serum from convalescent SARS-CoV-2 PCR positive patients or NHS (PCR negative) followed by anti-human IgG Alexa fluor 647 antibodies. Significant high levels of IgG binds to S were observed on the surface of HEK T293 cells.

To analyse complement activation on SARS-CoV-2 S protein using a model that mimics the natural surface expression in cells infected with SARS-CoV-2, we employed transiently transfected HEK 293T cells. In this model, a transient high level of expression of S protein on the surface of HEK 293T cells was achieved after transfection with the mammalian expression vector pEVAC containing the coding sequences for SARS-CoV-2 S protein. Cells expressing viral protein were incubated with NHS or pooled serum from convalescent patients, followed by detection of bound human antibodies by incubation with goat anti-human IgG Alexa fluor 647 antibodies. Fluorescence intensity was measured with The Attune NxT Flow Cytometer (Invitrogen). The level of anti-S protein antibody bound to HEK 293T cells was approximately 100 fold higher when using convalescent human serum compared to non-immune NHS (**Figure 5**). In a parallel experiment, C1q binding to HEK293 cells expressing S protein was not observed when using NHS (data not shown).

### Inhibition of LP Impairs Complement C3b Deposition on SARS-CoV-2 Proteins

**Figure 6:**
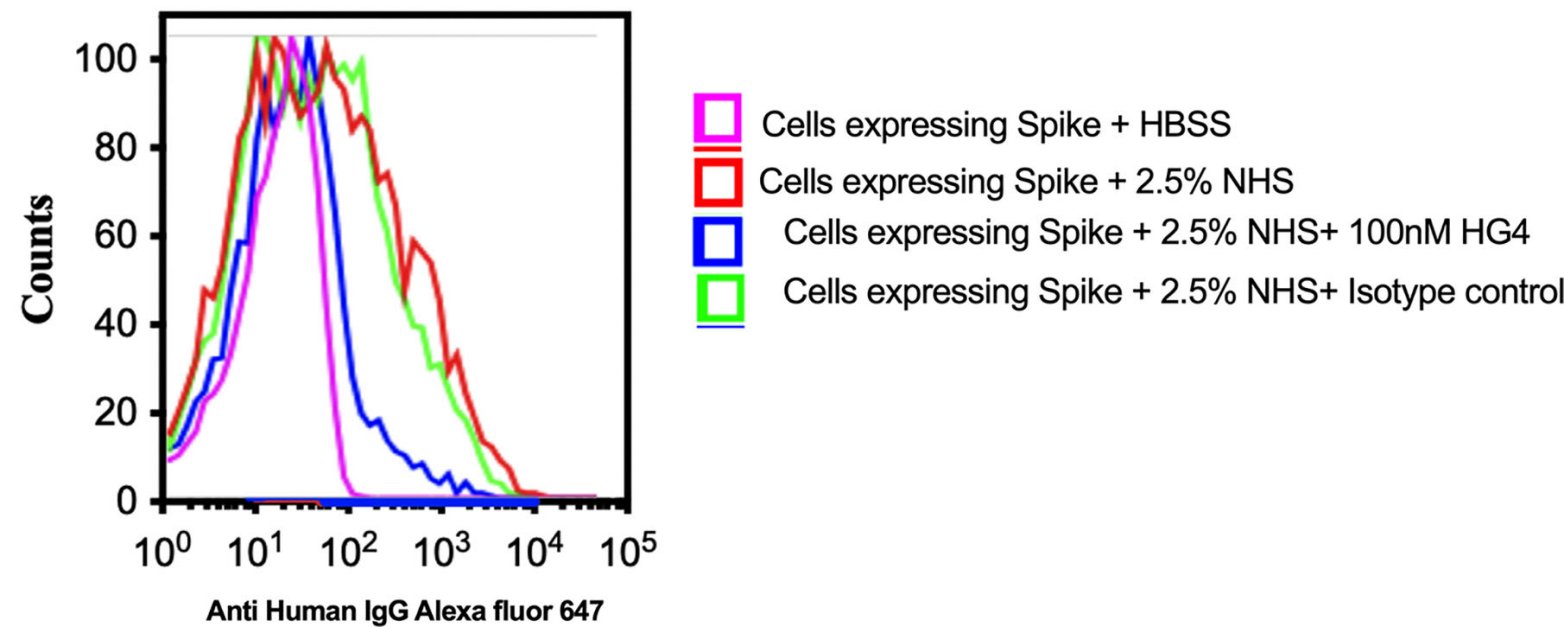
Inhibition of LP impairs complement C3b deposition on SARS-CoV-2 surface proteins. HEK 293T cells expressing SARS-CoV-2 S protein were used. Cells were incubated with 2.5% NHS with 100nM HG4 or an isotype control antibody at 37C for 30 min. Cells were washed and C3b deposition was detected using rabbit anti human C3b followed by goat anti rabbit FITC labelled antibodies. A significant C3b deposition was detected on HEK 293T cells expressing S protein. Inhibition of MASP-2 using HG4 significantly impairs complement C3b deposition on the surface of HEK 293T cells expressing S protein.

To evaluate complement C3b deposition on the surface of HEK 293T cells expressing S protein, cells were incubated with 2.5% NHS, and complement C3b deposition was detected using FACS analysis. A significantly higher level of complement C3b deposition was detected on cells expressing S protein compared to non-transfected cells. Inhibition of lectin pathway using HG4 significantly decreased complement C3b deposition from NHS (**Figure 6**).

## Discussion

SARS-CoV-2 is an emerging virus with a very high infectivity that causes life-threatening complications with mild to severe long-term morbidity and mortality, especially in patients with underlying medical conditions (16). The immunopathology differentiating mild from severe disease is not as yet sufficiently well understood to identify the windows of therapeutic opportunities for treatment (13, 17, 18). Excessive activation of complement, initiated in part by viral invasion of endothelial cells, causes collateral tissue injury (12). This work demonstrates that the LP recognition molecules MBL, FCN-2 and CL-11 bind to S and N proteins of SARS-CoV-2 with subsequent activation of LP-mediated C3b and C4b deposition. These findings clearly show the activation of the LP on SARS-CoV-2 surface proteins and N protein, confirming the central role of LP activation in the immunopathogenesis of COVID-19. Tissue damage consistent with complement-mediated microvascular injury has been observed in the lung and/or skin of patients with severe COVID-19, with significant deposition of the LP effector enzyme MASP-2, a hallmark of profound activation of the LP (14). Furthermore, extensive deposition of MASP-2 in the capillaries and venules of small bowel thrombotic microvascular injury in COVID-19 has also been reported, and endothelial complement staining patterns colocalized with staining of SARS-CoV-2 membrane and spike proteins (19). Our work also shows direct binding of MASP-2 to the N protein of SARS-CoV-2 with subsequent LP-mediated C4 cleavage into C4b and C4a, confirming the previous finding of Gao et al., who reported direct activation of MASP-2 on the SARS-CoV-2 N protein and showed that MASP-2-deficient mice are protected from disease (20). Since we used a truncated zymogen form of MASP-2, containing the CCP1, CCP2 and serine protease domains, our results also narrow down the N-protein binding site to these C terminal domains. Our *in vitro* study confirms that inhibition of MASP-2 blocks complement activation *via* the LP. Interestingly, Narsoplimab, a fully humanized immunoglobulin gamma 4 (IgG4) monoclonal antibody against MASP-2 that inhibits LP functional activity, has been used successfully in treatment of critically ill, mechanical ventilation-dependent COVID-19 patients. Patients who received Narsoplimab recovered and survived, demonstrating corresponding improvement/normalization of laboratory markers of inflammation (21). Many other complement inhibitors and anti-inflammatory drugs have been re-purposed and evaluated in COVID-19 clinical trials but, to date, none of those other agents have yielded a breakthrough in the treatment of severe COVID-19 (22–27). While vaccination is reducing hospitalisation, there is a fear of possible reduction in the efficacy against the new variants, which are responsible for severe cases of COVID-19 in younger age groups. Therapeutic approaches utilizing passive immunity (e.g., convalescent plasma, mono- and polyclonal antibodies), have also been disappointing, demonstrating no meaningful efficacy in severe COVID-19 and, possibly, adding to selection pressure on SARS-CoV-2. There remains a need to pursue aggressively and apply therapeutic strategies that block the COVID-19 immunopathological events and endothelial pathology, all of which appear to remain consistent across viral variants, with the objective of increasing access to any effective treatment(s) for acute as well as long-term post-COVID pathology. As an alternative to tackling the virus itself, protection from emerging new mutant viral strains could be achieved by tackling the immune physiological events that turn SARS-CoV-2 infections into generalised endothelial disease and ARDS in those susceptible to moderate-to-severe COVID-19. More research is needed to fully understand the disease processes triggered by SARS-CoV-2 at a molecular level.

The present study provides new insights into the direct triggers by SARS-CoV-2 at the protein level of LP activation in the early phase of COVID-19, and the role of the LP in types of long-haul COVID-19 are currently under investigation.

## Data Availability Statement

The original contributions presented in the study are included in the article/supplementary material. Further inquiries can be directed to the corresponding author.

## Author Contributions

YA and MF designed and performed the experiments. YA, MF, NL, JH, and WS wrote and revised the manuscript. SY, TD, SG, and GD provided essential reagents and revised the manuscript. All authors contributed to the article and approved the submitted version.

## Funding

This work was supported by National Institute for Health Research Grant G107217, awarded to WS.

## Conflict of Interest

WS, NL, and YA are consultants to Omeros Inc., which is developing inhibitors of the lectin pathway. GD, TD, SG, and SY are employed by Omeros Inc.

The remaining authors declare that the research was conducted in the absence of any commercial or financial relationships that could be construed as a potential conflict of interest.
